# The Influence of Perceived Organizational Support on Evidence-Based Practice among Jordanian Nurses: A Cross-Sectional Study

**DOI:** 10.1177/23779608251407928

**Published:** 2025-12-22

**Authors:** Sahar Nabeel Yousef, Ghada Abu-Shosha, Islam Ali Oweidat, Abdulqadir J. Nashwan, Kamaruddeen Mannethodi, Asma Ziadeh

**Affiliations:** 1 Faculty of Nursing, 108634 Zarqa University, Zarqa, Jordan; 2 Clinical Nursing Department, Faculty of Nursing, 108634 Zarqa University, Zarqa, Jordan; 3 Community and Mental Health Nursing Department, Faculty of Nursing, 74476 Zarqa University, Zarqa, Jordan; 4 Nursing and Midwifery Research Department, 36977 Hamad Medical Corporation, Doha, Qatar

**Keywords:** organizational support, evidence-based practice, nursing, healthcare, autonomy, decision-making, nurse empowerment, patient outcome, Jordan

## Abstract

**Introduction:**

Evidence-based practice (EBP) is vital for improving healthcare quality and patient outcomes, yet its successful implementation often depends on organizational factors such as perceived organizational support (POS). Nurses, as frontline healthcare providers, require both professional and organizational support to translate evidence into practice effectively. In Jordan, despite recognition of EBP's importance, its implementation among nurses faces challenges, with organizational support playing a pivotal role.

**Objectives:**

This study aimed to examine the association between POS and EBP implementation and to identify the predictors of EBP practice, attitudes, and knowledge among nurses in Jordan.

**Methods:**

A cross-sectional descriptive correlational design was employed. Data were collected from 188 registered nurses working in three major governmental hospitals in Jordan. POS was measured using the Perceived Organizational Support Scale, while EBP implementation was assessed using the Evidence-Based Practice Questionnaire. Descriptive statistics, Pearson's correlation, and multivariate regression analyses were conducted to determine relationships and predictors.

**Results:**

The findings revealed low-to-moderate levels of POS among participants (*M* = 17.52, SD = 3.78). For EBP, nurses reported moderate attitudes (*M* = 9.44, SD = 2.92) and knowledge/skills (*M* = 33.06, SD = 7.80), but relatively weak implementation in practice (*M* = 14.45, SD = 3.79). POS total score significantly correlated with EBP practice and implementation (*r* = .30, *P* = .002), EBP attitude (*r* = .81, *P* < .001), and EBP knowledge and skills (*r* = .50, *P* = .006). Age, participation in decision-making, autonomy in clinical practice, gender, and POS total score were significant predictors of EBP implementation. Female nurses and those reporting higher autonomy and organizational support demonstrated greater EBP engagement.

**Conclusion:**

The findings of this study empirically demonstrate that fostering an organizational culture of support—particularly by enhancing nurse autonomy and involvement in decision-making—is a critical lever for bridging the knowledge-to-practice gap in EBP.

## Introduction

Evidence-based practice (EBP) is widely recognized as a fundamental approach to improving patient outcomes, enhancing the quality of care, and optimizing organizational efficiency. The adoption of EBP has been linked to substantial improvements in patient outcomes and cost efficiency, reinforcing its role in creating more effective and sustainable healthcare systems (Alqahtani et al., 2022; Skyvell Nilsson et al., 2024). EBP entails the conscientious, explicit, and judicious use of current best evidence in making decisions about the care of individual patients (Sackett et al., 1996). In nursing, EBP has proven instrumental in improving patient outcomes by elevating the quality of healthcare, enhancing patient satisfaction, and reducing healthcare costs (Wang et al., 2023).

Nurses, often at the forefront of healthcare delivery, play a pivotal role in translating research into practice (Karadas et al., 2023; Yoo et al., 2019). However, the successful implementation of EBP is contingent upon various factors, including organizational support, access to resources, and ongoing professional development (Eisenberger et al., 1986; Ruzafa-Martínez et al., 2024). Studies consistently indicate that while nurses generally have positive attitudes toward EBP, they struggle to apply it consistently in their daily work, particularly in low- and middle-income settings. Some common barriers they face include limited access to training, a shortage of time and resources, and a lack of support from their organizations and management (Ruzafa-Martínez et al., 2024; Yoo et al., 2019).

Perceived organizational support (POS) is a crucial determinant in the successful implementation of EBP, as it reflects the extent to which employees feel valued and supported by their organization (Li-Hui et al., 2021). According to the social exchange theory (SET), the employees who feel supported by the organization are likely to be more committed and perform better (Eisenberger et al., 1986). POS encompasses providing the necessary resources, fostering professional development, and cultivating a supportive work environment (Karadas et al., 2023; Skyvell Nilsson et al., 2024). Structured job training is a vital form of support within organizations. Research shows that it plays a crucial role in enhancing employee performance, especially in the primary healthcare services sector (Prisia et al., 2024). In various global contexts, the presence of robust POS has been associated with increased nurse engagement, job satisfaction, and the successful application of EBP methodologies (Alqahtani et al., 2022; Ma’moun & Abu-Moghli, 2020). In addition, organizational support significantly impacts nurses’ job satisfaction and professional efficacy, which in turn influences their engagement with EBP (Ruzafa-Martínez et al., 2024; Wang et al., 2023).

Although the relationship between POS and EBP has been studied, little is known about these dynamics in the public health system in the Middle East, particularly Jordan, which has unique challenges. In Jordan, despite nurses exhibiting satisfactory levels of EBP knowledge and positive attitudes, the actual implementation rates remain low, highlighting a gap between knowledge and practice (Al-Hamdan & Bani Issa, 2022; Ma’moun & Abu-Moghli, 2020). The successful adoption of EBP largely depends on POS, which includes having adequate resources, strong leadership, proper education, and a culture that truly values evidence-based care (Li-Hui et al., 2021; Yoo et al., 2019). In Jordan's governmental hospitals, the lack of organizational and managerial support has been associated with nurse dissatisfaction, increased turnover intentions, and suboptimal patient outcomes (Al-Hamdan & Bani Issa, 2022). The limited availability of EBP training and resources continues to foster outdated clinical practices, preventable complications, and inefficiencies in patient care (Ruzafa-Martínez et al., 2024; Skyvell Nilsson et al., 2024). These systemic issues underscore the pressing need to explore how POS impacts EBP implementation, which could guide organizational and policy changes aimed at strengthening nursing capabilities, improving care quality, and aligning Jordan's public healthcare system with global standards. In addition, in Jordan, there is a notable gap between nurses’ knowledge of EBP and its actual implementation. Research has highlighted that Jordanian nurses might have the requisite knowledge and attitudes toward EBP, the lack of sufficient organizational support acts as a barrier to its effective implementation (Al-Hamdan & Bani Issa, 2022; Yoo et al., 2019). This gap between knowledge and practice indicates a critical need for healthcare organizations in Jordan to enhance POS by fostering a culture that prioritizes EBP, provides the necessary resources, and offers opportunities for continuous professional development (Abu-Baker et al., 2021; Fernández-Castro et al., 2023).

## Review of Literature

Several studies have explored the role of organizational factors in influencing EBP implementation among nurses. Li-Hui et al. (2021) found that Taiwanese nurse practitioners who perceived higher organizational support demonstrated greater job satisfaction and engagement in EBP. Similarly, Karadas et al. (2023) reported that POS enhanced psychological resilience and EBP implementation among Turkish nurses, particularly during the COVID-19 pandemic. Skyvell Nilsson et al. (2024) emphasized that a positive organizational culture and managerial support reduced stress and ethical dilemmas, improving nurses’ engagement with EBP in Sweden.

In China, Wang et al. (2023) identified a positive relationship between POS, professional self-esteem, and the effective application of EBP, suggesting that supportive work environments directly contribute to nurses’ clinical performance and decision-making. Ruzafa-Martínez et al. (2024) similarly highlighted that targeted EBP training and institutional support significantly improved EBP competencies among Spanish nurses. Conversely, Yoo et al. (2019) found that despite positive attitudes toward EBP among South Korean nurses, inadequate organizational readiness and resource limitations hindered consistent EBP application.

In the Middle Eastern context, Al-Hamdan and Bani Issa (2022) demonstrated significant associations between POS, work engagement, and self-efficacy among Jordanian nurses, while Ma’moun and Abu-Moghli (2020) observed moderate levels of EBP knowledge and practice, largely constrained by insufficient institutional backing. Abu-Baker et al. (2021) also found that EBP training improved beliefs and implementation among nursing students, underscoring the value of educational and organizational investment in EBP promotion.

Despite these insights, few empirical studies have specifically examined the relationship between POS and EBP implementation within Jordan's governmental hospitals. Existing research often focuses on knowledge and attitudes without fully exploring how organizational factors facilitate or hinder actual EBP practice. Therefore, this study aims to fill this gap by examining the levels of POS and EBP implementation, exploring their relationship, and identifying predictors of EBP practice among Jordanian nurses.

## Methods

### Research Design

This study used descriptive correlational, cross-sectional research design. The descriptive design allowed the researcher to assess the levels of POS and EBP implementation among nurses in Jordanian governmental hospitals at a specific point in time, capturing their manifestation within the sample under study (Polit & Beck, 2021).

### Study Setting

The study was conducted across three Jordanian governmental hospitals as given below, which were selected for the diversity of services they offer, providing a broad context to investigate POS and EBP among nurses.

- Al Bashir Hospital, established in 1956, offers primary, secondary, and tertiary care services and has 1,256 beds and 1,437 nurses and midwives. It is the largest governmental hospital in Jordan, treating around 50,000 patients monthly.
- New Zarqa Governmental Hospital, located in the Zarqa Governorate, is a modern facility capable of handling a large patient turnout and is equipped with advanced healthcare technologies, contributing to enhanced patient outcomes.
- Prince Faisal Governmental Hospital, also in Zarqa, has 195 beds and provides comprehensive healthcare services to meet the region's healthcare demands.

### Participants

The target population consisted of all registered nurses practicing in the selected Jordanian hospitals. Eligibility criteria included nurses of various ages, genders, educational levels, and those with at least 1 year of experience in direct patient care. Nurses in managerial or nonnursing positions and those not directly involved in patient care were excluded from the study.

### Sample Size

The sample size was calculated using G*Power software version 3.1.9.7 (Faul et al., 2009), based on multiple regression analysis with the following parameters: *F*-test, linear multiple regression: fixed model, *R*^2^ deviation from zero, a power of 0.80, alpha = .05, and a medium effect size of 0.15, considering 13 predictors (e.g., POS, age, gender, education level, clinical autonomy). A minimum sample size of 131 participants was required, but the sample size was increased to 200, accounting for nonresponse.

### Data Collection

Data collection began after receiving the required ethical and administrative approvals. The primary researcher visited the hospital administrators and nurse managers seeking permission to gain access to participants. A convenience sampling method was used to recruit participants from the selected hospitals. Potential participants were approached by the primary researcher with assistance from the nurse managers in each respective department. Participants who met the eligibility criteria were invited to participate. The primary researcher explained the study purpose, risks, and benefits, and obtained informed consent electronically. An online survey was used for data collection, distributed via email and WhatsApp. The online survey was developed from the survey engine which makes it easy to access and complete by the participants. Participants were encouraged to complete the survey that was in English within 10 days. Reminders were sent and support from nursing managers helped motivate timely completion of the survey. The data collection period spanned 5 weeks, from May 23rd to June 30th of 2024.

### Ethical Considerations

Ethical approval for this study was obtained from the Faculty of Nursing at Zarqa University (No. 14/2023), and the selected hospitals (No. 7778/19/5/2024). Informed consent was secured from each participant after explaining the purpose of the study. Participation was voluntary and participants had the right to refuse or withdraw from the study at any time. Participant anonymity and confidentiality were ensured through coded data and restricted researchers-only access. No identifiable information was collected.

### Instruments

#### Sociodemographic and Work-Related Variables

A Sociodemographic and Work-Related Variables Data Sheet was used to gather information on participants’ age, gender, years of practice, educational level, department, shift schedule, and prior EBP training. Other variables included involvement in organizational decision-making, perceived autonomy in practice, and perceived resources for EBP implementation.

#### Perceived Organizational Support Scale

The Perceived Organizational Support Scale (POSS), developed by Kraimer and Wayne (2004), was used to measure POS among nurses. The scale includes 12 items across three domains: career support, financial support, and adjustment (the latter excluded in this study as it applies to expatriates). Participants rated each item on a 5-point Likert scale (1 = strongly disagree, 5 = strongly agree), with scores categorized as low (<50%), moderate (50%–75%), or high (>75%; Abo Habieb & Elwkeel, 2020). The reliability coefficient (Cronbach's alpha) for this tool was .89, indicating high reliability (Abo Habieb & Elwkeel, 2020).

#### Evidence-Based Practice Questionnaire

The Evidence-Based Practice Questionnaire (EBPQ), developed by Upton and Upton (2006), was used to assess nurses’ attitudes, knowledge, and use of EBP. The EBPQ consists of 24 items, measured on a 7-point Likert scale (1 = never, 7 = always), divided into three subscales: practice of EBP (6 items), attitude toward EBP (4 items), and knowledge/skills of EBP (14 items). The EBPQ has demonstrated robust psychometric properties with high internal consistency (alpha = .87) and construct validity supported by significant correlations with EBP awareness (Upton & Upton, 2006).

### Data Analysis

Data analysis was conducted using SPSS version 21, with the significance level set at .05. Descriptive statistics were used to summarize sample characteristics and examine levels of POS and EBP. Pearson correlation analysis was used to assess the relationships between POS, demographic characteristics, and EBP implementation. Multivariate regression was used to determine the significant predictors of EBP implementation, including factors such as POS, education, and years of experience. Assumptions for statistical tests were thoroughly checked. For descriptive statistics, data normality was assessed using frequency distribution plots, Shapiro–Wilk tests, and *Q*–*Q* plots. For Pearson correlation analysis, the normality of data (*Q*–*Q* plots) and homoscedasticity (scatter plots, residual plots) were evaluated. Assumptions for multivariate linear regression, including linearity, independence of errors (Durbin–Watson statistic), and the absence of multicollinearity by reporting the variance inflation factor value, and homoscedasticity (residual plots), were rigorously tested.

## Results

Out of 200 surveys distributed, 188 were returned, resulting in a response rate of 94%. Half of the participants were males and age ranges from 24 to 52 years. The experience of the study participants ranged from 2 to 30 years. The detailed demographic and work characteristics are given in Table 1. Participation in decision-making had a mean score of 2.15 (SD = 0.42) on a 1–5 scale. The perception of autonomy in clinical practice had a mean score of 2.15 (SD = 1.20), while the perception of resources available for implementing EBP and frequency of opportunities to engage in EBP activities both had a mean score of 2.19 (SD = 1.15 and SD = 1.17, respectively).

**Table 1.** Sociodemographic and Work-Related Characteristics of the Study Participants.

Variable	Category	Frequency (%)
Age	Mean ± SD = 31.53 ± 6.08	
Experience	Mean ± SD = 9.53 ± 4.18	
Gender	Male	94 (50.0)
	Female	94 (50.0)
Educational level	Bachelor	150 (79.8)
	Postgraduates	38 (20.2)
Department	Medical/surgical	70 (37.2)
	Critical care	40 (21.3)
	Pediatrics	39 (20.7)
	Obstetrics/gynecology	39 (20.7)
Shift	Day shift	54 (28.7)
	Night shift	80 (42.6)
	Rotating shifts	54 (28.7)
Engagement in continuous Education/training	Yes	53 (28.2)
	No	135 (71.8)
Previous exposure to training on EBP	Yes	64 (34.0)
	No	124 (66.0)

EBP = evidence-based practice.

### Reliability Tests

In order to evaluate the scales’ internal consistency, Cronbach's alpha for each individual scale and subscales was calculated. The reliability coefficient (Cronbach's alpha) for the POSS was .79; for career subscale, .78; for financial subscale, .73; and for adjustment subscale was .81. The reliability coefficient for the EBPQ was .83; for the practice of EBP, .77, for the attitude toward EBP, .84; and for knowledge/skills associated with EBP, .79. All scales showed acceptable values, and therefore, items have high internal consistency.

### The Levels of POS and the Extent of EBP Implementation

The levels of POS and the extent of EBP implementation are shown in Table 2. The mean score of POS score is 17.52 (SD = 3.78).

**Table 2.** Levels of POS and the Extent of EBP Implementation.

Variable	Mean	SD	Minimum	Maximum
POS	17.52	3.78	11	36
Career support	8.80	3.05	4	20
Financial support	8.72	2.55	4	16
Practice of EBP	14.45	3.79	7	27
Attitude toward EBP	9.44	2.92	4	19
Knowledge/skills of EBP	33.06	7.80	18	59

POS = Perceived organizational support; EBP = evidence-based practice.

The frequency analysis of POS levels showed that 58.5% of nurses had low POS scores, 34.5% had moderate scores, and 7.0% had high POS scores (see Table 3).

**Table 3.** Frequency Analysis for Levels of POS Among Nurses.

POS Level	Frequency	Percent
Low	110	58.5
Moderate	65	34.5
High	13	7.0

POS = Perceived organizational support.

### Relationship Between POS and EBP Implementation

Table 4 presents the Pearson correlation results examining the relationship between POS and EBP implementation among nurses. The POS total score showed significant positive correlations with all three EBP subscales: EBP practice and implementation (*r* = .30, *P* = .002), EBP attitude (*r* = .81, *P* ≤ .001), and EBP knowledge and skills (*r* = .50, *P* = .006).

**Table 4.** Correlations Between POS and EBP Implementation Subscales.

Variables	EBP Practice and Implementation	EBP Attitude	EBP Knowledge and Skills
Financial subscale	*r* = .139* (*P* = .008)	*r* = .89* (*P* = .027)	*r* = .35** (*P* = .009)
Career subscale	*r* = .69* (*P* = .015)	*r* = .66* (*P* = .035)	*r* = .31 (*P* = .077)
POS total score	*r* = .30* (*P* = .002)	*r* = .81** (*P* = .00)	*r* = .50* (*P* = .006)

POS = Perceived organizational support; EBP = evidence-based practice.

*Correlation is significant at the .05 level (2-tailed), **correlation is significant at the .01 level (2-tailed).

### Predictors of EBP Implementation

A regression analysis was performed to identify predictors of EBP implementation. The predictors included age, participation in decision-making processes, perception of autonomy, perception of resources, gender, educational level, shift type, and the total POS score.

### Predictors of EBP Practice and Implementation

The regression model shows a significant overall fit with an *R* value of .678, indicating a strong correlation between the predictors and the outcome variable. The *R*^2^ value of .611 suggests that approximately 61.1% of the variance in EBP implementation and practice is explained by the predictors included in the model. The adjusted *R*^2^ value is slightly lower at .554, accounting for the number of predictors and the sample size (*R*^2^ = .611, adjusted *R*^2^ = .554, *F*(7, 180) = 18.125, *P* < .000). Details of the regression model are presented in Table 5. Age is a significant positive predictor, indicating that older nurses are more likely to implement EBP. Participation in decision-making processes and perception of autonomy in clinical practice are also significant positive predictors, suggesting that nurses who are more involved in decision-making and perceive higher autonomy are more likely to practice EBP. The perception of resources available for implementing EBP is another significant predictor. Gender, is a significant predictor, indicating that female nurses are more likely to implement EBP. The POS total score is also a significant predictor, highlighting the importance of POS in promoting EBP implementation among nurses.

**Table 5.** Predictors of EBP Practice and Implementation.

Variable	*B*	Beta	*t*	*P*	95% Confidence Interval
Age	1.09	0.37	6.36	.00	[0.75 to 1.43]
Years of experience	0.14	0.01	0.20	.81	[−1.23 to 1.51]
Participation in decision-making	0.38	0.29	3.33	.03	[0.16 to 0.60]
Perception of autonomy	0.44	0.10	1.76	.00	[−0.05 to 0.93]
Perception of resources for EBP	0.25	0.12	0.82	.02	[−0.35 to 0.85]
Gender	1.92	0.12	1.71	.00	[−0.28 to 4.12]
Educational level	2.23	0.17	1.43	.09	[−0.83 to 5.29]
Shift type	2.70	0.15	1.32	.05	[−1.31 to 6.71]
POS total score	3.24	0.15	2.19	.03	[0.34 to 6.14]

EBP = evidence-based practice; POS = perceived organizational support.

## Discussion

This study explored the relationship between POS and EBP implementation among nurses in Jordanian governmental hospitals. The findings indicate that POS plays a crucial role in shaping nurses’ attitudes, knowledge, and practice of EBP.

### Levels of POS and EBP Implementation

The study revealed generally low levels of POS among the participants, with mean scores of 8.72 for the financial subscale and 8.80 for the career subscale. These low POS levels could reflect a lack of adequate organizational support in Jordanian hospitals, potentially hampering nurses’ engagement in EBP. Previous research, such as Skyvell Nilsson et al. (2024), similarly highlighted that a positive organizational culture improves the ethical environment and reduces stress among nurses, thereby facilitating EBP implementation.

In contrast, the nurses demonstrated moderately positive attitudes toward EBP and possessed strong knowledge and skills related to EBP, with a mean knowledge score of 33.06. However, the actual practice and implementation of EBP scored lower, indicating a significant gap between knowledge and its application in clinical settings. This trend is consistent with Li-Hui et al. (2021), who found that while POS improved job satisfaction and care outcomes, EBP implementation remained suboptimal without adequate institutional support. The findings of the current study and those of Li-Hui et al. (2021) can be understood through the unique organizational dynamics that affect nurses’ engagement in EBP.

These findings are important because POS directly affects nurses’ ability to engage in EBP. SET supports this connection, suggesting that when nurses perceive strong organizational backing, they are more likely to invest in EBP (Eisenberger et al., 1986). In nursing, this means that when hospitals offer sufficient resources, recognition, and chances for professional development, nurses feel more inspired to use evidence in their clinical decision-making. A positive organizational support not only builds commitment but also boosts nurses’ confidence and sense of effectiveness, helping them tackle complex clinical situations while sticking to EBP principles. This connection is especially important in non-Western contexts, where strict hierarchies and limited autonomy can make it tough to turn knowledge into action. Karadas et al. (2023) also emphasized that organizational support fosters resilience among nurses, further enhancing their capacity to implement EBP, especially in high-stress situations like the COVID-19 pandemic. Therefore, having managerial support, access to resources, and recognizing the contributions of nurses helps in translating knowledge into practice.

### Relationship Between POS and EBP Implementation

This study found significant positive correlations between POS subscales and EBP implementation. For instance, the POS financial subscale was positively correlated with EBP knowledge and skills (*r* = .35, *P* = .009) and EBP attitude (*r* = .89, *P* = .03). This suggests that financial support, including adequate remuneration and resources, helps foster both positive attitudes and improved EBP knowledge. These findings align with Wang et al. (2023), who demonstrated that organizational support enhances self-esteem and professional efficacy, leading to better engagement with EBP. The similarities found in these studies may stem from common mechanisms, whereby support provided by the organization—whether it is through financial backing, recognition, or opportunities for professional growth—enables nurses to effectively incorporate evidence into their clinical practice.

Moreover, the POS career subscale showed significant correlations with EBP practice and implementation, indicating that career supports such as opportunities for promotion and professional development encourages nurses to integrate EBP into their practice. This supports Al-Hamdan and Bani Issa (2022), who also found that POS boosts work engagement and self-efficacy in Jordanian hospitals, with higher levels of support linked to better professional outcomes. The similarities in these studies can be linked to a shared underlying factor; when nurses feel that their growth and professional development are appreciated by their organization, they are more likely to translate knowledge and positive attitudes into actual practice.

Taken together, these results reinforce the importance of fostering organizational support to enable the successful implementation of EBP. In healthcare systems where POS is high, nurses are more likely to apply EBP consistently, improving patient care and operational efficiency. Conversely, inadequate support can lead to burnout and lower engagement, as demonstrated by Yoo et al. (2019), who found that despite positive EBP attitudes, South Korean nurses struggled to implement EBP due to insufficient organizational readiness.

### Predictors of EBP Implementation and Practice

Several key predictors of EBP implementation emerged in this study, including age, participation in decision-making, autonomy in clinical practice, and gender. Older nurses were more likely to practice EBP, potentially due to their longer exposure to EBP principles and accumulated experience. This finding is consistent with adult learning theory, which suggests that adults apply new knowledge more effectively as they age (McGrath, 2009). These findings suggest that having professional maturity is essential for successfully implementing EBP.

Participation in decision-making was another significant predictor, indicating that when nurses are empowered to contribute to decisions about patient care, they are more motivated to engage with EBP. This aligns with Kanter's theory of structural empowerment, which posits that employees who participate in decision-making feel a greater sense of ownership and responsibility for their work outcomes. Autonomy also significantly predicted EBP implementation, reflecting the importance of self-directed practice in fostering evidence-based care, as supported by self-determination theory (Laschinger et al., 2001). Beyond its role as a positive work characteristic, autonomy can also be interpreted as a modern power mechanism (Velásquez, 2025). When nurses are given the freedom to make their own decisions, it promotes self-regulation and helps them align with the goals of the organization. This means that autonomy not only fosters professional development but also guides nurses’ actions in a way that supports the organization's priorities, ultimately encouraging the use of EBP. These insights highlight how structural empowerment and autonomy serve as both valuable resources and tools that organizations use to influence practice behaviors, showcasing the vital connection between organizational culture and evidence-based nursing.

Interestingly, female nurses were more likely to engage in EBP than their male counterparts. This finding echoes Wang et al. (2023), who noted that female nurses tend to take on more education and development roles, which may contribute to their stronger engagement with EBP. The similarity can be attributed to the fact that female nurses often take the initiative to pursue learning and skill development. This proactive approach leads to a deeper understanding of EBP principles and a stronger commitment to applying evidence-based interventions. However, years of experience, educational level, and shift type were not significant predictors, suggesting that organizational support and empowerment play a more critical role in fostering EBP than individual factors alone. The nonsignificance of years of experience could be attributed to the fact that experience does not equal EBP unless it is complemented by continuing education and organizational promotion. Likewise, the nonsignificance of educational level and shift type could suggest that while these variables are significant, they might not be as dominant when organization support and empowerment strategies are in place. This interpretation supports the idea that organizational culture and support can potentially override individual characteristics, creating an environment where all nurses, regardless of experience or education level, are encouraged and enabled to implement EBP.

These findings are consistent with Karadas et al. (2023), who showed that organizational support is a key determinant of resilience and EBP implementation among nurses. They also align with Li-Hui et al. (2021), who found that POS and job satisfaction positively correlated with EBP implementation among Taiwanese nurses, emphasizing the global relevance of these predictors. This suggests that these mechanisms work in various cultural and healthcare settings. Altogether, these findings highlight the worldwide importance of POS as a crucial factor in successfully implementing EBPs.

### Strengths and Limitations

This study contributes several strengths to the literature on POS and EBP. It is one of the few empirical studies conducted within Jordanian governmental hospitals—an understudied context—thereby providing important regional insights. The high response rate (94%) enhances the reliability of the findings, and the use of well-validated instruments (POSS and EBPQ) strengthens the methodological rigor and supports comparability with international studies.

Despite these strengths, few limitations must be acknowledged. First, the use of a cross-sectional descriptive design limits the ability to establish causality between POS and EBP implementation. The findings therefore reflect associations rather than directional effects. Second, reliance on self-reported measures may introduce response bias, as participants may have overestimated or underestimated their levels of organizational support or EBP engagement. The absence of objective or observational measures reduces the ability to verify whether perceived practices align with actual clinical behaviors. Additionally, although the sample size was adequate, the use of convenience sampling limits the representativeness of the findings and affects generalizability beyond the selected hospitals.

Incorporating these limitations, several directions for future research emerge. Longitudinal studies are recommended to examine the long-term influence of POS on EBP implementation across different healthcare contexts. Interventional studies, particularly quasi-experimental designs, could assess the effectiveness of leadership training programs for nurse managers aimed at strengthening POS and evaluating subsequent impacts on EBP. Furthermore, qualitative research exploring nurses’ lived experiences and perceptions, especially in high-acuity environments such as intensive care units would help identify deeper contextual barriers and facilitators of EBP engagement. Extending research across various healthcare sectors and geographic regions would further validate and broaden the applicability of the current findings.

### Implications for Practice

The findings of this study highlight the need for healthcare organizations to actively foster environments that support nurses in adopting EBP. Enhancing POS should be seen not only as a key focus for managers but also as a vital health-promoting strategy, as insufficient support and heavy workloads have been shown to negatively affect nurses’ mental well-being. Practical strategies to enhance nursing practice include setting up structured career development and mentorship programs, acknowledging and rewarding nurses who excel in EBP, and ensuring there is dedicated time and resources for EBP initiatives. By empowering nurses to take part in decision-making and offering leadership training for nurse managers can further strengthen professional autonomy and foster a culture of continuous learning. By addressing these organizational factors, nursing managers can strengthen professional autonomy, build positive attitudes toward EBP, and increase nurses’ confidence in applying evidence to clinical care. In turn, this not only enhances the quality and safety of patient outcomes but also contributes to greater job satisfaction, resilience, and retention of nursing staff. These results emphasize that EBP cannot be fully realized without supportive organizational structures, and thus, investments in supportive policies and leadership strategies are essential.

## Conclusion

This study provides valuable insights into the relationship between POS and EBP implementation among nurses in Jordanian governmental hospitals. The results indicate that POS—particularly in terms of financial and career support—plays a significant role in fostering positive EBP attitudes, knowledge, and practice. Factors such as age, decision-making involvement, autonomy, and gender were also significant predictors of EBP implementation. The findings support the SET, which posits that organizational support encourages employees to reciprocate with higher levels of engagement and performance, including EBP implementation (Eisenberger et al., 1986). Moreover, the study highlights the importance of fostering empowerment and autonomy in clinical practice to ensure that nurses can effectively implement EBP. From these findings, healthcare organizations are encouraged to enhance work environments by providing adequate resources, promoting autonomy, and engaging nurses in decision-making processes. Such strategies will not only strengthen EBP implementation but also improve job satisfaction and nurse retention.
